# Dual promoters of the major catalase (KatA) govern distinct survival strategies of *Pseudomonas aeruginosa*

**DOI:** 10.1038/srep31185

**Published:** 2016-08-05

**Authors:** In-Young Chung, Bi-o Kim, Hye-Jeong Jang, You-Hee Cho

**Affiliations:** 1 Department of Pharmacy, College of Pharmacy and Institute of Pharmaceutical Sciences, CHA University, Gyeonggi-do, 13488, Korea

## Abstract

KatA is the major catalase required for hydrogen peroxide (H_2_O_2_) resistance and acute virulence in *Pseudomonas aeruginosa* PA14, whose transcription is driven from the promoter (*katAp1*) located at 155 nucleotide (nt) upstream of the start codon. Here, we identified another promoter (*katAp2*), the +1 of which was mapped at the 51 nt upstream of the start codon, which was responsible for the basal transcription during the planktonic culture and down-regulated upon H_2_O_2_ treatment under the control by the master regulator of anaerobiosis, Anr. To dissect the roles of the dual promoters in conditions involving KatA, we created the promoter mutants for each -10 box (*p1m, p2m*, and *p1p2m*) and found that *katAp1* is required for the function of KatA in the logarithmic growth phase during the planktonic culture as well as in acute virulence, whereas *katAp2* is required for the function of KatA in the stationary phase as well as in the prolonged biofilm culture. This dismantling of the dual promoters of *katA* sheds light on the roles of KatA in stress resistance in both proliferative and growth-restrictive conditions and thus provides an insight into the regulatory impacts of the major catalase on the survival strategies of *P. aeruginosa*.

*Pseudomonas aeruginosa* is a non-fermentative bacterial pathogen that respires on oxygen as well as nitrogen oxides, and its mode of respiration is likely associated with the infection strategies: *P. aeruginosa* is capable of rapid colonization primarily through aerobic respiration during acute infections, which involve various virulence factors^1^; in contrast, *P. aeruginosa* survives persistently as a result of chronic infections promoted by biofilm mode of growth, which involve microaerobic or anaerobic respiration on nitrate (NO_3_^−^) under the hypoxic condition within the microbial population^2^. Thus, the characteristics of both infection strategies that *P. aeruginosa* exploits are quite distinct and known to be associated with a subset of virulence or persistence factors that should be orchestrated to support the survival of *P. aeruginosa* during each mode of interaction with the host environments^3^. For example, invasive functions such as secreted toxins and adhesion factors are required for acute virulence, but detrimental for chronic infection or persistence, depending on their cost-benefits^4^. This is demonstrated by accumulation of the mutations in the invasive functions over time, once infection is established^5^.

We previously reported the role of the major catalase, KatA in peroxide resistance, osmoprotection, and acute virulence of a *P. aeruginosa* strain^6^. Unlike other major bacterial catalases, KatA is highly stable and found in the extracellular milieu, which ensures the survival of *P. aeruginosa* cells in their biofilm^7^. The basal expression of KatA is relatively high, but its expression is further increased upon stationary growth phase and in response to H_2_O_2_ under the control of OxyR, the master regulator in response to H_2_O_2_. OxyR normally binds to the upstream region of the *katA* promoter as a functional repressor and switches to the activator upon H_2_O_2_ challenge^8^,^9^, where C199 has a critical role in both functions and in acute virulence^10^. Recently, KatA was shown to play a critical role in nitric oxide buffering under anaerobic respiration conditions, upon which KatA expression was increased by Anr, the master regulator of anaerobiosis required for dissimilatory NO_3_^−^ reduction^11^. It has been suggested by Trunk *et al.*^12^ that KatA may belong to the Anr regulon, with a presumable Anr box located upstream of the KatA initiation codon. Although much has been being unveiled about the physiological roles of KatA and the regulatory mechanisms that involve OxyR and its *cis*-element of the *katA* promoter, more needs to be clarified to account for the involvement of Anr and the elevated expression of KatA upon anaerobic respiration for its roles to ensure the distinct infection strategies of *P. aeruginosa*.

In the present study, we have explored the regulatory module underlying the anaerobic regulation of KatA, by newly identifying a second promoter (*katAp2*) of the *katA* gene, which is responsible for the basal expression of KatA during the planktonic growth of *P. aeruginosa* strain PA14. This second promoter is regulated primarily by Anr. More importantly, the mutation analyses of each promoter revealed the separate roles of the dual promoters to cope with reactive oxygen and/or nitrogen species (ROS and RNS), which is dependent on the growth states.

## Results

### Identification of a second promoter (*katAp2*) of the *katA* gene

**Figure 1:**
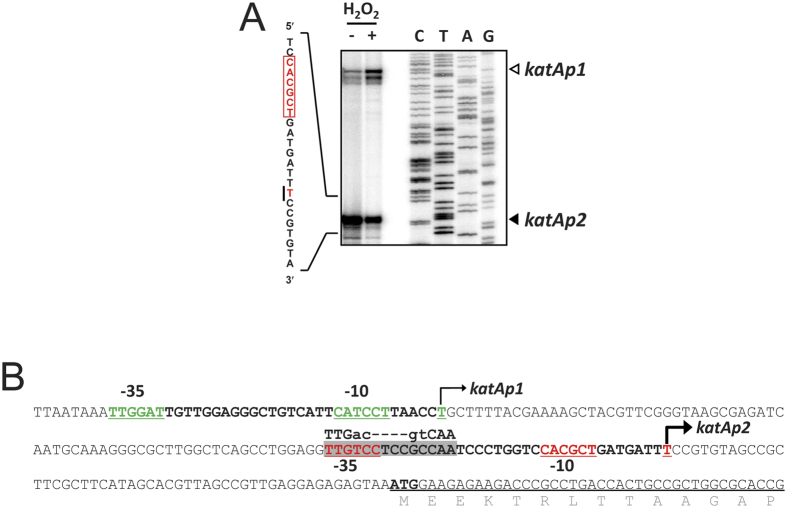
Promoter region of the *katA* gene. (**A**) High-resolution S1 nuclease mapping. The 5′ end of the *katA* mRNA transcript was determined by high-resolution S1 nuclease mapping. Total RNA (50 μg) that had been prepared from the cells with (+) or without (−) 1 mM H_2_O_2_ treatment for 10 min at OD_600_ of 1.0 in LB were subjected to S1 nuclease mapping. The sequencing reactions (lanes C, T, A and G) were carried out as described in Methods. The two transcriptional start sites of the *katA* gene are indicated by open (*katAp1*) and closed (*katAp2*) arrows. The detailed transcriptional start site of *katAp2* promoter is shown on the left by solid line, and its putative −10 box is enclosed in box. (**B**) Promoter elements of the *katA* gene. The sequence of the *katA* promoter region of the *katA* gene is shown, with the two 5′ ends of *katAp1* and *katAp2* promoters indicated by the bent arrows. The Anr binding consensus (TTGac-N4-gtCAA) is designated, whose center is located at −29 position from the *katAp2* promoter. The putative −10 and −35 boxes are underlined. The N-terminal amino acid sequences of the *katA* gene are designated.

A transcription start site of the *katA* gene was identified at the 155 nucleotide (nt) upstream (T-155) of the *katA* initiation codon, whose transcription is induced upon H_2_O_2_ treatment. Despite over 5-fold induction of *katAp1* transcription upon H_2_O_2_ exposure, the *katA-lacZ* fusion displayed a minor (~2-fold) induction^8^, which may be attributed to another level of regulation. Based on this speculation, we have identified another promoter of the *katA* gene, *katAp2*: from the high-resolution S1 nuclease mapping data, a second protective band was observed at the 51 nt upstream (T-51) of the translation initiation codon (Fig. 1A). The upstream of the position of T-51 could be a potential promoter by its context. It is of marked interest that the putative Anr box consensus (TTGac-N_4_-gtCAA) was identified, which overlaps with the potential −35 box of *katAp2* (Fig. 1B), since KatA expression is deemed reduced in the *anr* mutant as previously assessed by the transcriptome and proteome analyses^12^. Identification of the *katAp2* promoter and the potential Anr box suggests that the *anr*-dependent regulation of KatA might be most likely attributed to this second promoter. Moreover, the *katAp2* transcript was slightly reduced upon H_2_O_2_ treatment (Fig. 1A; see below), which is in a good agreement with the fact that the Fe-S center of Anr is vulnerable to ROS and RNS with its transactivation activity compromised in the presence of O_2_^−^, H_2_O_2_, and NO^13^,^14^,^15^.

### Regulation of *katAp2* by Anr during aerobic NO_3_^−^ respiration

**Figure 2:**
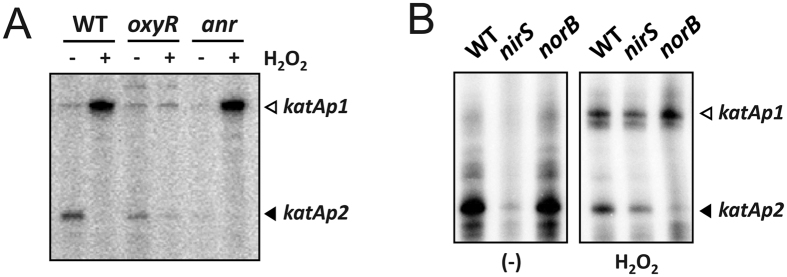
Transcription profiles of *katA* promoters in mutants. The transcription patterns were assessed by low-resolution S1 nuclease protection assay with H_2_O_2_ treatments in *oxyR* and *anr* mutants grown in LB (**A**) and in *nirS* and *norB* mutants grown in LB amended with 15 mM KNO_3_ (**B**). Total RNA (50 μg) that had been prepared from the wild type (WT) and the mutant (*oxyR*, *anr*, *nirS*, and *norB*) cells with (+) or without (−) 1 mM H_2_O_2_ treatment for 10 min at OD_600_ of 0.5 were subjected to S1 nuclease assay. The two transcriptional start sites of the *katA* gene are indicated by open (*katAp1*) and closed (*katAp2*) arrows.

To verify the involvement of Anr in the regulation of *katAp2*, we examined the *katA* transcription in the *anr* null mutant, by using low-resolution S1 nuclease protection assay (Fig. 2A). The *katAp1* transcription was highly elevated, whereas the *katAp2* transcription was slightly reduced upon H_2_O_2_ treatment in the wild type bacteria. In contrast, however, the *katAp2* transcription in the *anr* mutant was significantly reduced even in the absence of H_2_O_2_ treatment and the *katAp1* transcription was not affected in the *anr* mutant. Furthermore, the H_2_O_2_-mediated decrease in *katAp2*-driven transcription is not associated with OxyR, in that the *katAp2* transcription pattern in the *oxyR* null mutant did not significantly differ from that in the wild type bacteria. This result suggests that the dual promoters of *katA* were regulated separately by two global regulators, OxyR and Anr.

Since the *katAp2* transcription is abolished in the *anr* mutant, accounting for the basal expression of KatA during the planktonic growth, we have tested if the Anr-dependent NO_3_^−^ respiration during the planktonic growth may be associated with the *katAp2* transcription as well. We have exploited the mutants for the genes encoding another downstream regulator, Dnr and the four nitrogen oxide reductases (Nar, Nir, Nor, and Nos) involved in dissimilatory denitrification^16^: NO_3_^−^ is reduced to nitrite (NO_2_^−^) by Nar; NO_2_^−^ is reduced to NO by Nir; NO is reduced to N_2_O by Nor; N_2_O is reduced to N_2_ by Nos. Denitrification is vital for growth and survival under microaerobic and anaerobic conditions as found in biofilms and microcolonies.

We found that only the *nirS* mutant displayed reduced *katAp2* transcription (Figs 2B and S1). This is in good agreement with the previous observation by Su *et al.*^11^ that NO and Anr might be required for the increased KatA activity under anaerobic conditions.

### Creation of the *katA* promoter mutants

**Figure 3:**
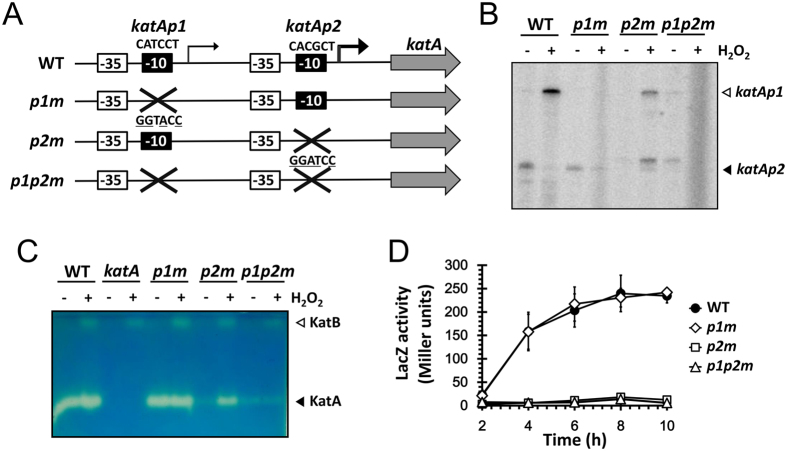
Creation of the *katA* promoter mutants. (**A**) Schematic representation. The *katA* gene and its potential promoter elements (−35 and −10 boxes) are designated. The sequences of each −10 box are indicated above the boxes. The *katA* promoter mutations were constructed by substituting the −10 box with the *Kpn*I site (GGTACC) for the *katAp1* mutant (*p1m*) or the *Bam*HI site (GGATCC) for the *katAp2* mutant (*p2m*) as indicated with the mutated nucleotides underlined. A double mutant for both promoters (*p1p2m*) was generated as well. (**B**) Transcription upon H_2_O_2_ induction. Total RNA (50 μg) isolated from the wild type (WT) and the *katA* promoter mutant (*p1m*, *p2m*, and *p1p2m*) cells with (+) or without (−) 1 mM H_2_O_2_ treatment for 10 min at OD_600_ of 0.5 in LB were subjected to low-resolution S1 nuclease assay. The two transcriptional start sites of the *katA* gene are indicated by open (*katAp1*) and closed (*katAp2*) arrows. (**C**) Catalase activity staining upon H_2_O_2_ induction. Catalase activities in cell extracts of the wild type (WT) and the *katA* null and promoter mutant (*katA*, *p1m*, *p2m*, and *p1p2m*) cells with (+) or without (−) 1 mM H_2_O_2_ treatment for 10 min as in (**B**) were visualized using 50 μg of proteins in each cell extract. The two catalase bands are indicated by open (KatB) and closed (KatA) arrows. (**D**) Promoter activity during aerobic planktonic growth. The wild type cells harboring one of the promoter transcription fusions (•, WT; ◇, *p1m*; □, *p2m*; ∆, *p1p2m*) were grown in LB amended with 15 mM KNO_3_. Culture aliquots were taken at every 2 h from 2 to 10 h post-inoculation and then subjected to β-galactosidase (LacZ) assay. The data represent the average of the means of three independent experiments (two cultures per experiment), with the error bars representing the standard deviations.

To explore the roles of the dual promoters in the expression of KatA that is required for several phenotypes such as stress resistance and virulence in *P. aeruginosa*^6^, we created the promoter mutants for each presumable −10 box, as described in Fig. 3A. The substitution mutations were introduced into the chromosome by pEX18T-based allelic exchange, which was confirmed by PCR followed by restriction enzyme digestions. The functional verification of the created promoter mutants (*p1m, p2m and p1p2m*) was performed based on the *katA* transcription and catalase activity profiles in the mutants (Fig. 3B,C). The *katAp1* and *katAp2* transcripts from the promoter mutants were analyzed by S1 nuclease protection assay, which verified that the introduced substitutions at the proposed −10 box resulted in the specific loss of the corresponding transcripts. A slower migrating band that was newly observed by the *p2m* mutation did not affect the catalase profiles in the mutants (Fig. 3C). As a result, the basal expression of KatA during the planktonic growth was abolished by the *p2m* mutation throughout the entire growth phases (Fig. 3D), whereas the *p1m* mutation contributed only to the induced expression upon H_2_O_2_ treatment, which was more evident in the *p2m* mutant (Figs. 3C and S2). These results indicate that the *katAp2* promoter is involved in the basal as well as increased expression during the planktonic growth, whereas *katAp1* is responsible for the H_2_O_2_-inducible expression of KatA. Because the *katAp2* requires the transactivator, Anr for its transcription, we have postulated that Anr is somehow active even in the aerobic planktonic culture in complex media such as PIA that contains ~63 μM NO_3_^−^ used for NO_3_^−^ respiration^17^ (Fig. 2). However, as shown in Fig. S3, the stationary phase-induced transcription of *katAp2* was not completely abolished in the double mutant for *anr* and *rpoS*, indicating that it involves more complicated regulatory networks such as quorum sensing and so on^18^.

### *katAp1* is critical in the well-growing state, whereas *katAp2* is critical in the growth-restricting state.

**Figure 4:**
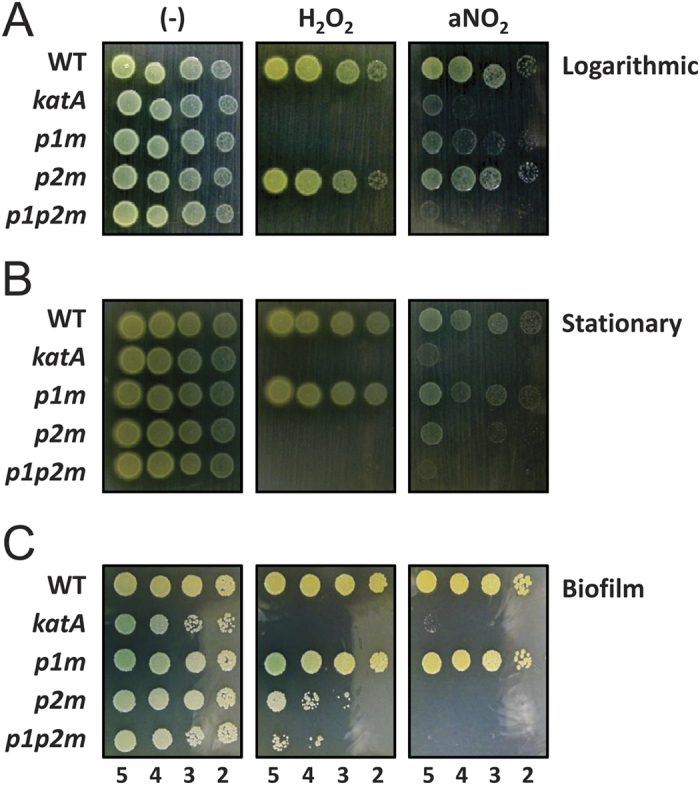
Stress resistance of the *katA* promoter mutants. (**A**,**B**) Stress resistance in planktonic culture. Susceptibility to H_2_O_2_ and acidified NaNO_2_ (aNO_2_) were assessed for the wild type (WT) and the *katA* null and promoter mutant (*katA*, *p1m*, *p2m*, and *p1p2m*) cells that had been cultured for 3 h or for 6 h in LB broth (pH 6.5), and treated for 24 h with either 15 mM H_2_O_2_ and 200 μM NaNO_2_ or in LB broth (pH 6.5) (−). Ten-fold serial cell dilutions from the 3-h (**A**; logarithmic) and 6-h (**B**; stationary) cultures were spotted on LB agar plate. The numbers indicate the log (cfu) of the applied bacterial spots. (**C**) Stress resistance in static culture. The wild type (WT) and the *katA* null and promoter mutant (*katA*, *p1m*, *p2m*, and *p1p2m*) cells were statically and anaerobically cultured for 7 days in LB broth (pH 6.5) treated for 1 h with either 100 mM H_2_O_2_ and 1.2 M NaNO_2_ or in LB broth (pH 6.5) (−). Ten-fold serial cell dilutions from the cultures were spotted on LB agar plate. The numbers indicate the log(cfu) of the applied bacterial spots.

We first investigated the several stress-related phenotypes of the promoter mutants in comparison with the *katA* null mutant, which is hypersusceptible to H_2_O_2_ or acidified nitrite (aNO_2_)^6^,^11^,^19^. As shown in Fig. 4A, when the cells were tested during the logarithmic growth phase, the *p1m* mutant and the *p1p2m* mutant were hypersusceptible to H_2_O_2_ or aNO_2_ treatment, whereas the *p2m* mutant was no more susceptible than the wild type. In contrast, however, when cells were taken during the stationary growth phase or from the biofilm culture, the outcome did clearly differ: the *p2m* mutant as well as the *p1p2m* mutant was hypersusceptible to H_2_O_2_ or aNO_2_ treatment, whereas the *p1m* mutant was no more susceptible than the wild type (Fig. 4B,C). These results may be associated with the inducible expression of KatA by *katAp1* when the basal expression of KatA by *katAp2* might be insufficient, and also with the higher basal expression of KatA by *katAp2* upon the stationary growth phase when the stress-induced expression of KatA through *katAp1* is likely compromised in this general stress condition^20^.

### *katAp1,* but not *katAp2*, contributes to acute virulence.

**Figure 5:**
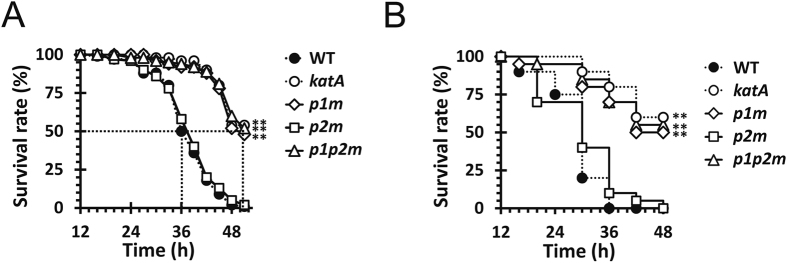
Acute virulence of the *katA* promoter mutants. Virulence in *Drosophila* (**A**) and mouse peritoneal (**B**) infection models. The wild type (WT) and the mutant (*katA*, *p1m*, *p2m*, and *p1p2m*) cells were prepared and the survival of infected animals was determined as described in Methods. The values are the averages from five replicate experiments for *Drosophila* (**A**) and three for mouse (**B**) infections. Statistical significance based on the log-rank test is indicated (**p < 0.005).

We next measured the virulence potential of the promoter mutants using *Drosophila* and murine acute infection models, since the *katA* null mutant of PA14 strain was virulence-attenuated in the same infection models^6^. It is of marked interest that *p1m* and *p1p2m*, but not *p2m* displayed virulence attenuation in both infection models (Fig. 5), suggesting that the inducible KatA expression driven by *katAp1*, is critical in the host environments. Although we do not have any direct evidence on whether the *katAp2* transcription would occur during the infection conditions, it is clear that the *katAp2*-mediated KatA expression is likely dispensable in the virulence pathways at least under our experimental conditions.

### *katAp2* contributes to lowering endogenously generated RNS.

**Figure 6:**
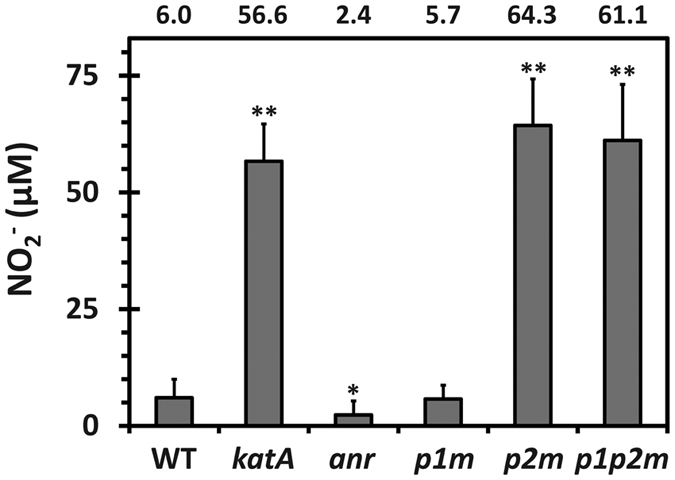
Accumulation of NO_2_^−^ in the *katA* promoter mutants. Steady-state level of extracellular NO_2_^−^ was measured in the wild type (WT) and the *katA* null and promoter mutant (*katA*, *p1m*, *p2m*, and *p1p2m*) cells as well as *anr* mutant cells that had been grown to OD_600_ of 0.7. The amount (μM) of NO_2_^−^ is calculated using the standard curve (*r*^2^ = 0.999) and the average values measured from the three independent experiments, with the error bars representing the one positive value of the standard deviations. Statistical significance based on the Student’s *t*-test is indicated (*p < 0.01; **p < 0.005).

We hypothesized that KatA might have some surveillance function for homeostatic maintenance of cellular states regarding ROS and RNS by lowering the levels of endogenously generated ROS and RNS. We speculated that ROS and/or RNS could be accumulated in the *p2m* mutant, because *katAp2* is required for the basal expression of KatA throughout the entire growth phases (Fig. 3D). We were able to measure the steady-state level of NO_2_^−^ accumulated during the planktonic growth in LB broth supplemented with 15 mM NO_3_^−^, which can be used as an alternative electron acceptor through dissimilatory NO_3_^−^ reduction involving three consecutive intermediates (NO_2_^−^, NO, and N_2_O) to the end product (N_2_)^16^. Among the intermediates in the denitrification process, NO is highly unstable and vulnerable to spontaneous oxidation to NO_2_^− ^21. Therefore, the amount of NO_2_^−^ (either periplasmic or extracellular) may reflect the degree of NO_3_^−^ respiration and the concomitant level of periplasmic RNS including NO^22^. Figure 6 shows that the *katA* null mutant as well as the *katAp2* mutants (*p2m* and *p1p2m*) exhibited significantly higher levels (56.6~64.3 μM) of NO_2_^−^ than the wild type and the *p1m* mutant (5.7~6.0 μM) during the planktonic growth. This result indicates the role of KatA as well as its *katAp2*-driven expression to lower the endogenous generation of RNS, under assumption that the NO_3_^−^ respiration activity of the *katA* mutant does not significantly differ from that of the wild type bacteria, which requires more extensive investigation.

## Discussion

KatA is the major catalase of *P. aeruginosa*, whose “adaptive” function is to dismutate H_2_O_2_, an ROS either sporadically generated during the normal respiration on O_2_ or externally provided by redox-cycling agents or host factors. However, KatA exhibited higher activity under anaerobic conditions than aerobic conditions^23^, where no ROS could be potentially generated. This “enigmatic” expression profile of KatA under anaerobic conditions has been granted with the evidence that the KatA expression is lower in the *anr* mutant than in the wild type, with the potential Anr box located upstream of the *katA* coding region^12^. However, it had not been fully substantiated until the study about KatA function in anaerobiosis was reported^11^. They found that KatA contributes to the resistance to aNO_2_ that generates RNS like NO. They also proposed that KatA binds to NO to sequester and prevent it from its harmful effect, a new role of KatA in buffering free NO to ensure *P. aeruginosa* anaerobiosis, based on the investigation of direct NO-KatA interaction under anaerobic condition, as first demonstrated in bovine liver catalase able to complex with NO at the heme^24^. Although the anaerobic KatA expression could be justified by its proposed function, the exact regulatory mechanisms have remained elusive. In this study, we first connect the dots between the previous results regarding the expression and the function of KatA under anaerobic conditions, by identifying the second promoter, *katAp2*. This promoter is positively regulated by Anr and required to lower the endogenous generation of RNS during planktonic culture, which we now call the “surveillance” function of KatA.

In our previous study, *P. aeruginosa* KatA exhibits unusual properties ascribed to its metastability, high specific activity, and/or extracellular presence, unlike the other clade 3 monofunctional bacterial catalases such as *Streptomyces coelicolor* CatA and *Bacillus subtilis* KatA (BsKatA)^7^. At least one of those properties has been proposed to be implicated in biofilm life style of *P. aeruginosa. P. aeruginosa* respires on NO_2_^−^ and/or NO_3_^−^ under anaerobic conditions, which is in marked contrast to the two bacterial species: *S. coelicolor* is unable to grow under anaerobic conditions; *B. subtilis* is capable of fermentation under anaerobic conditions. Interestingly, BsKatA is subjected to activity modulation called “instant adaptation” by NO in response to ROS^11^,^25^. Although the careful comparison between KatA and the related catalases needs to be furthered in regards to the physiological traits of the cognate bacteria, the functions of KatA in the microaerobic and anaerobic life style of *P. aeruginosa* may be attributed to the unusual biochemical properties of KatA, most likely the extracellular presence, as previously suggested^7^. This aspect will open a new venue for an insight into molecular evolution of a clade 3 bacterial catalase to fulfill the new needs that the bacterium is faced with, by delving into the roles of the particular amino acid residues of KatA associated with the extracellular and/or periplasmic presence and the protective function to buffer the NO, because NO is normally generated in the periplasm by Nir during during microaerobic and/or biofilm growth of *P. aeruginosa*^16^.

Another important contribution of this work is to provide a new picture to associate the protective functions of KatA with its regulation governed by OxyR and Anr. It should be noted that OxyR and Anr work apparently under the opposite conditions: for example, OxyR is activated by H_2_O_2_ or NO^26^, while Anr is inactivated by H_2_O_2_ or NO, because its Fe-S cluster is vulnerable to H_2_O_2_ and NO^13^,^14^,^15^. Nevertheless, the dual promoters ensure the *katA* transcription in both balanced (i.e. surveillance function-requiring) and perturbed (i.e. adaptive function-requiring) conditions of ROS and/or RNS homeostasis. It is also likely that OxyR and Anr may cooperate for proper KatA expression during infection, due to the complicated respiration mode for *P. aeruginosa* growth that would be microaerophilic along the continuum between aerobic and anaerobic conditions in the human airways^27^,^28^.

Finally, the present study clearly demonstrates the discrete roles of the dual promoters in the KatA-associated phenotypes. The creation of the chromosomal mutations for the presumable −10 boxes worked well to disrupt each promoter activity and the subsequent KatA expression specifically from the corresponding promoters. Use of these promoter mutants helped clarify the roles of each promoter in the functions of KatA in *P. aeruginosa* physiology. Based on these properties regarding regulation and function, the pivotal roles of KatA during the growth and survival of *P. aeruginosa* warrant further verification. This can be done by using the promoter mutants created in the present study. Moreover, the complex involvement of multiple regulatory systems will be comprehensively explored at various regulatory levels, especially in response to stationary phase, quorum-sensing, and/or iron availability etc. All these aspects that will be more directly elucidated may provide a new insight into the therapeutic targets of this and the related bacteria associated with the stress responses toward ROS and RNS.

## Methods

### Bacterial strains and culture conditions

The bacterial strains and plasmids used in this study are listed in Table 1. *E. coli* and *P. aeruginosa* strains were grown at 37 °C using Luria-Bertani (LB) broth or on 1.5% Bacto-agar solidified LB plates. For anaerobic growth, bacteria were grown in LB medium supplemented with 15 mM KNO_3_ in an anaerobic jar with AnaeroPack (MGC). Overnight-grown cultures were used as inoculum (1.6 × 10^7 ^cfu/ml) into fresh LB broth and grown at 37 °C to the OD_600_ as indicated and then used for the experiments described herein.

### DNA oligonucleotide primers

The information on DNA oligonucleotide primers used for gene deletion, expression, and detection in this study are listed in Table S1.

### RNA isolation and S1 nuclease protection analysis

*P. aeruginosa* strains were grown in LB or LB containing 15 mM KNO_3_ media and then the half of the culture was treated with 1 mM H_2_O_2_ for 10 min, with the remaining half used as the untreated control. Total RNA was isolated from 10^9^ cells by using RNeasy kit (Qiagen) and 50 μg of RNA samples were used for S1 nuclease protection experiment as described elsewhere^8^. Briefly, the PCR-generated probe using the oligonucleotide primer pairs (katA-N10: 5′ end at −133 and katA-S1C1: 5′ end at +264) was labeled with [γ-^32^P] ATP by T4 polynucleotide kinase. For hybridization, the mixtures of RNA samples and labeled probe were incubated at 90 °C for 10 min for denaturation, and then slowly cooled down to 55 °C for hybridization. After overnight hybridization, S1 nuclease digestion was performed at 30 °C for 30 min by adding 8 units of S1 nuclease for each sample. The reaction was stopped and precipitated with 100% ethanol. The samples were dissolved and denatured at 90 °C for 5 min in formamide-dye solution, and then analyzed by 6% PAGE containing 7 M urea. For high resolution S1 mapping, the unlabeled katA-S1C1 was used to generate the nucleotide sequence ladder using Sequenase Version 2.0 DNA Sequencing Kit (USB) with [α-^32^P] dATP and pUCP-*katA* as the template^7^.

### Creation of the *katA* promoter mutants

The *katA* promoter mutant allele for the *katAp1* (−10 box; CATCCT to GGTACC, the KpnI site) or for the *katAp2* (−10 box; CACGCT to GGATCC, the BamHI site) were generated by SOEing (splicing by overlap extension) PCR using 4 oligonucleotide primers. The resulting PCR products were cloned into pEX18T. These *katA* promoter mutant alleles were introduced into PA14 chromosome as described elsewhere^29^.

### Creation of the *lacZ* fusions and β-galactosidase assay

All the *lacZ* fusions of the *katA* promoter regions were created by SOEing PCR using 4 oligonucleotide primers (Table S1). The primers were designed to generate transcriptional fusions of the *katA* promoter regions without its own ribosome-binding site (RBS) at the upstream of the *lacZ* RBS. Briefly, the *katA* promoter regions were prepared by using the primers pairs (katA-N3 and katA-lacZ-UC) and each of the chromosomes from the WT and the promoter mutant (*p1m*, *p2m* and *p1p2m*) bacteria as the templates. The *lacZ* coding region was generated by using the primer pairs (katA-lacZ-DN and pQF50-lacZ-C1) and the pQF50 plasmid as the template. The PCR products were fused by SOEing PCR using katA-N3 and pQF50-lacZ-C1 primers and the amplified fragments were cloned into pQF50. These promoter fusion constructs were introduced by electroporation and LacZ (β-galactosidase) activity was determined using the bacterial culture aliquots taken at the indicated time points as previously described^30^.

### Catalase activity staining

Catalase activity staining was performed as described previously^7^. Briefly, cell extracts (40 μg) were applied to a 7% native polyacrylamide gel and electrophoresed. The gel was washed in distilled water and then treated with 1 mM H_2_O_2_ for 10 min. After treatment, the gel was rinsed and transferred to 1% (w/v) ferric chloride and 1% (w/v) potassium ferricyanide solution. The reaction was stopped by washing in distilled water.

### Stress susceptibility measurement

Stress susceptibility was measured based on the survival of the *P. aeruginosa* cells upon 24 h treatment to 15 mM H_2_O_2_ or 200 μM NaNO_2_ for prolonged exposure. For pulse-treatment, cells were exposed to 100 mM H_2_O_2_ or 1.2 M NaNO_2_ in acidified LB (pH 6.5). After exposure, 10-fold serial dilutions (3 μl) of the cells were spotted onto an LB agar plate to enumerate the survivor bacteria.

### Virulence measurement

Determination of mortality from *Drosophila* systemic infections and mouse peritonitis-sepsis caused by *P. aeruginosa* cells that had been grown to the OD_600_ of 3.0 was performed as previously described^30^. For *Drosophila* systemic infections, 3- to 6-day-old adult female flies (Oregon R) were infected by pricking at the dorsal thorax with a 10 mm needle (Ernest F. Fullam, Inc.). The needle dipped into bacterial suspensions diluted in 10 mM MgSO_4_ containing 10^7^ cfu. Fly mortality was monitored for 54 h postinfection. For murine peritonitis-sepsis, bacterial cells were harvested, washed twice with phosphate buffered saline (PBS) (2.7 mM KCl, 137 mM NaCl, 10 mM Na_2_HPO_4_, and 2 mM KH_2_PO_4_, pH 7.0) and diluted to 2 × 10^6 ^cfu in 100 ml of PBS containing 1% mucin as an adjuvant. Anesthetized 4-week-old mice (ICR) were intraperitoneally infected according to the institutional protocol approved by the Institutional Animal Care and Use Committee at CHA University. Kaplan-Meier analysis and log-rank tests were used to compare the virulence difference between the groups^30^,^31^. A *p* value of less than 0.01 was considered to be significant.

### NO_2_^−^ measurement

The steady-state level of NO_2_^−^ that had been endogenously generated and transported during the normal growth in LB amended with 15 mM KNO_3_ was measured based on Griess reaction. Culture aliquots (300 μl) of the cells that had been grown to OD_600_ of 0.7 were mixed with the equal volume of Griess reagent (Sigma, USA) and incubated at 37 °C for 10 min. The NO_2_^−^ level in the supernatant was measured by the absorbance at 550 nm. Statistical significance between the groups is indicated, based on a *p* value of less than 0.01 by the Student’s *t* test.

## Additional Information

**How to cite this article**: Chung, I.-Y. *et al.* Dual promoters of the major catalase (KatA) govern distinct survival strategies of *Pseudomonas aeruginosa. Sci. Rep.* **6**, 31185; doi: 10.1038/srep31185 (2016).

## Supplementary Material

Supplementary Information

## Figures and Tables

**Table 1:** Bacterial strains and plasmids used in this study.

Strain or plasmid	Relevant characteristics or purpose^a^	Reference or source
*P. aeruginosa*		
PA14	wild type laboratory strain; Rif^R^	Lab collection
*katA*	PA14 with in-frame deletion of *katA*; Rif^R^	Lee *et al.*^6^
*oxyR*	PA14 with in-frame deletion of *oxyR*; Rif^R^	Choi *et al.*^32^
*anr*	PA14 with in-frame deletion of *anr*; Rif^R^	This study
*rpoS*	PA14 with in-frame deletion of *rpoS*; Rif^R^	Park *et al.*^33^
*anrrpoS*	*anr* with in-frame deletion of *rpoS*; Rif^R^	This study
*narG*	PA14 with *MAR2* × *T7* insertion at *narG*; Rif^R^, Gm^R^	Liberati *et al.*^34^
*nirS*	PA14 with *MAR2* × *T7* insertion at *nirS*; Rif^R^, Gm^R^	Liberati *et al.*^34^
*norB*	PA14 with *MAR2* × *T7* insertion at *norB*; Rif^R^, Gm^R^	Liberati *et al.*^34^
*nosZ*	PA14 with *MAR2* × *T7* insertion at *nosZ*; Rif^R^, Gm^R^	Liberati *et al.*^34^
*dnr*	PA14 with *MAR2* × *T7* insertion at *dnr*; Rif^R^, Gm^R^	Liberati *et al.*^34^
*p1m*	PA14 with the chromosomal mutation (CATCCT to GGTACC)^b^ at the −10 box of *katAp1*; Rif^R^	This study
*p2m*	PA14 with the chromosomal mutation (CACGCT to GGATCC)^b^ at the −10 box of *katAp2*; Rif^R^	This study
*p1p2m*	*p1m* with the chromosomal mutation (CACGCT to GGATCC)^b^ at the −10 box of *katAp2*; Rif^R^	This study
*E. coli*		
DH5α	multi-purpose cloning	Lab collection
S17-1	conjugal transfer of mobilizable plasmid; Tp^R^; Sm^R^	Lab collection
Plasmids		
pEX18T	Positive selection suicide vector for allelic exchange; Cb^R^	Lab collection
pEX18T-∆*anr*	pEX18T with the in-frame deletion in the *anr* gene; Cb^R^	This study
pEX18T-*katAp1m*	pUCP18 with the −10 box mutation (CATCCT to GGTACC)^b^ of *katAp1*; Cb^R^	This study
pEX18T-*katAp2m*	pUCP18 with the −10 box mutation (CACGCT to GGATCC)^b^ of *katAp2*; Cb^R^	This study
pQF50	*lacZ* transcriptional fusion; Cb^R^	Farinha and Kropinski^35^
pQF50-*katAp*	pQF50 with the *katA* promoter; Cb^R^	This study
pQF50-*katAp1m*	pQF50 with the −10 box mutation (CATCCT to GGTACC)^b^ of *katAp1*; Cb^R^	This study
pQF50-*katAp2m*	pQF50 with the −10 box mutation (CACGCT to GGATCC)^b^ of *katAp2*; Cb^R^	This study
pQF50-*katAp1p2m*	pQF50 with the −10 box mutation (CATCCT to GGTACC)^b^ of *katAp1* and (CACGCT to GGATCC)^b^ of *katAp2*; Cb^R^	This study

^a^Rif ^R^, rifampicin-resistant; Gm^R^, gentamicin-resistant; Cb^R^, carbenicillin- and ampicillin-resistant; Tp^R^, trimethoprim-resistant; Sm^R^, stremptomycin-resistant.

^b^underlines; mutated nucleotides at the presumable −10 boxes.
