# Supplementary material for: Dual promoters of the major catalase (KatA) govern distinct survival strategies of Pseudomonas aeruginosa
Source: Sci Rep. 2016 Aug 5;6:31185. doi: 10.1038/srep31185 (PMC4974557; doi:10.1038/srep31185)
Supplement: Supplementary Information [file srep31185-s1.pdf]

Supplementary Information

Dual promoters of the major catalase (KatA) govern distinct survival strategies of *Pseudomonas aeruginosa*

In-Young Chung, Bi-o Kim, Hye-Jung Jang, and You-Hee Cho*

Department of Pharmacy, College of Pharmacy and Institute of Pharmaceutical Sciences, CHA University, Gyeonggi-do 463-400, Korea

* Corresponding author.
Phone: 82-31-881-7165.
E-mail: youhee@cha.ac.kr

Running Title: Dual promoters for dual roles of KatA

Keywords: *Pseudomonas aeruginosa*, *katA*, H_2O_2, nitrogen oxides

Supplementary Information

Table S1. Primers used in this study.

Primer[a]	***Sequence (engineered enzyme site)***	***Purpose***
Gene mutation and S1 mapping		
anr-N1	5′-GCGTCGCGAATTCGAGCCC-3′ (*Eco*RI)	*anr* deletion
anr-C1	5′-GCCCAGCCCAGAAGCTTCCA -3′ (*Hind*III)	*anr* deletion
anr-UC	5′-ACTGCTGGGCGGAGAACAGCGAATCCATGTCT-3′	*anr* deletion
anr-DN	5′-AGACATGGATTCGCTGTTCTCCGCCCAGCAGT-3′	*anr* deletion
katA-N10	5′-CCGAATAAGGCATCTGCTGC-3′	*katAp* mutation/ S1 mapping
katA-S1C1	5′-ACCACACGTCCTGCAGCAAC -3′ (*Pst*I)	*katAp* mutation/ S1 mapping
katAp1m-UC	5′-CAGGTTAGGTACCAATGACAGCCCTCCAACAATC-3’ (*Kpn*I)′	*katAp1m* mutation
katAp1m-DN	5′- TGTCATTGGTACCTAACCTGCTTTTACGAAAAGC-3′(*Kpn*I)′	*katAp1m* mutation
katAp2m-UC	5′-AATCATCGGATCCGACCAGGGATTGGCGGAGGA-3′ (*Bam*HI)	*katAp2m* mutation
katAp2m-DN	5′-CCTGGTCGGATCCGATGATTTCCGTGTAGCC-3′ (*Bam*HI)	*katAp2m* mutation
LacZ transcriptional fusion		
katA-N3	5′-CGTAGATCTGGTTGATCGTC-3′ (*Bgl*II)	LacZ fusion
pQF50-lacZ-C1	5′-CAGCAGGATATCCTGCACC-3′ (*Eco*RV)	LacZ fusion
katA-lacZ-UC[b]	5′-TTAATACCCTCTAGCTAG**AACGTGCTATGAAGCGAA**-3′	LacZ fusion
katA-lacZ-DN[b]	5′-**TTCGCTTCATAGCACGTT**CTAGCTAGAGGGTATTAA-3′	LacZ fusion

a: N and C refer to the forward and reverse primers for each gene, respectively.

b: The *katA* promoter region is indicated (bold).

Supplementary Information

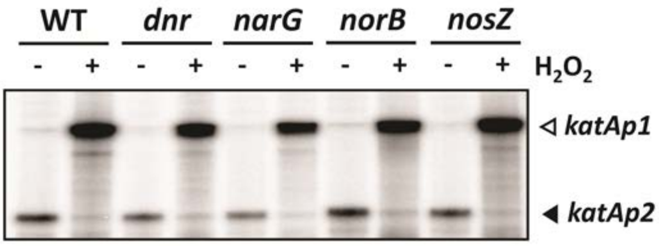

Fig. S1. Transcription profiles of *katA* promoters in various mutants.

The transcription patterns were assessed by low-resolution S1 nuclease assay with H_2O_2 treatments in some mutants for dissimilatory nitrate respiration grown in LB. Total RNA (50 µg) that had been prepared from the wild type (WT) and the mutant (*dnr*, *narG*, *norB*, and *nosZ*) cells with (+) or without (-) 1 mM H_2O_2 treatment for 10 min at OD_{600} of 0.5 were subjected to S1 nuclease assay. The two transcriptional start sites of the *katA* gene are indicated by open (*katAp1*) and closed (*katAp2*) arrows.

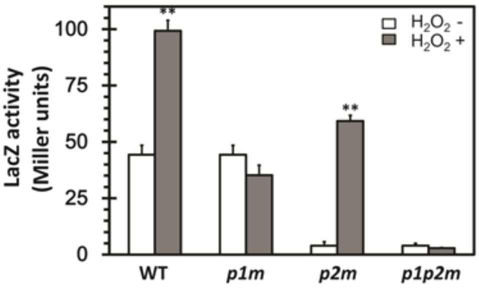

Fig. S2. H_2O_2-induced transcription of the *katA* promoter fusions.

H_2O_2-induced *katA* promoter activities were determined in the wild type cells containing one of the promoter fusions (WT, *p1m*, *p2m* and *p1p2m*). The cells were grown in LB with 15 mM KNO_3 to the mid-logarithmic growth phase, and then treated with (filled bar) or without (empty bar) 1 mM H_2O_2 for 10 min. The aliquots from the cells were subjected to β-galactosidase (LacZ) assay. The error bars represent the standard deviations from five independent experiments (two cultures per experiment). Statistical significance based on the Student's *t*-test is indicated (**, $p < 0.005$).

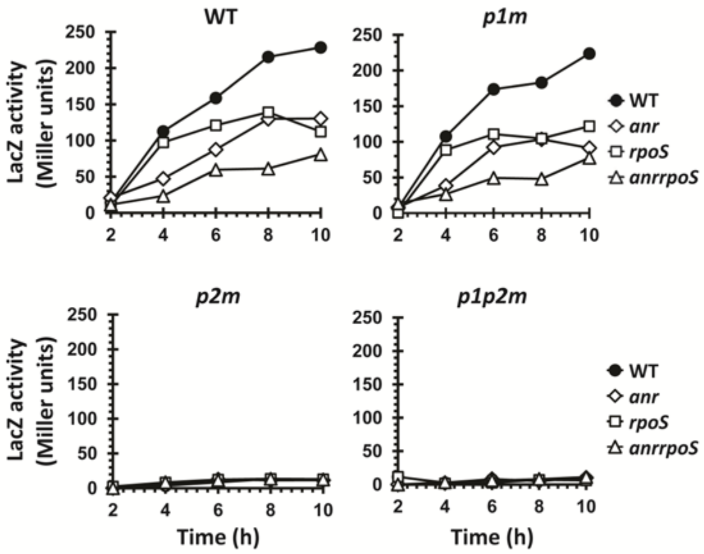

Fig. S3. Stationary phase-induced transcription of the *katA* promoter fusions. The growth phase-dependent transcriptions of the *katA* promoter fusions were assessed using PA14 (●, WT) as well as in its isogenic *anr* and *rpoS* null mutants (◇, *anr*; □, *rpoS*; △, *anr rpoS*), which harbor one of the *katA* promoter fusions (A, WT; B, *p1m*; C, *p2m*; D, *p1p2m*). The culture aliquots were harvested at every 2 h from 2 to 10 h post-inoculation, and then subjected to LacZ assay. The data represent the average of the means of three independent experiments (two cultures per experiment).
